# Retraction of Deficient LRRC8A-dependent volume-regulated anion channel activity is associated with male infertility in mice

**DOI:** 10.1172/jci.insight.195729

**Published:** 2025-06-23

**Authors:** Jianqiang Bao, Carlos J. Perez, Jeesun Kim, Huan Zhang, Caitlin J. Murphy, Tewfik Hamidi, Jean Jaubert, Craig D. Platt, Janet Chou, Meichun Deng, Meng-Hua Zhou, Yuying Huang, Héctor Gaitán-Peñas, Jean-Louis Guénet, Kevin Lin, Yue Lu, Taiping Chen, Mark T. Bedford, Sharon Y.R. Dent, John H. Richburg, Raúl Estévez, Hui-Lin Pan, Raif S. Geha, Qinghua Shi, Fernando Benavides

Original citation: *JCI Insight*. 2018;3(16):e99767. https://doi.org/10.1172/jci.insight.99767

Citation for this retraction: *JCI Insight*. 2025;10(12):e195729. https://doi.org/10.1172/jci.insight.195729

At the request of the co-corresponding author, *JCI Insight* is retracting this article. Concerns were previously raised regarding potential image duplications in Figures 2F and 5C. An institutional investigation could not verify authenticity of the results presented in these figures, as the original data were not available.

## Version 1 06/23/2025

Electronic publication

